# Greater Care Program in the face of the challenges of aging: a qualitative analysis

**DOI:** 10.11606/s1518-8787.2023057004859

**Published:** 2023-09-29

**Authors:** Cláudio Phillipe Fernandes de Castro, Janaína de Souza Aredes, Karla Cristina Giacomin, Josélia Oliveira Araújo Firmo

**Affiliations:** I Fundação Oswaldo Cruz Instituto René Rachou Programa de Pós-graduação em Saúde Coletiva Belo Horizonte MG Brazil Fundação Oswaldo Cruz. Instituto René Rachou. Programa de Pós-graduação em Saúde Coletiva. Belo Horizonte, MG, Brazil; II Fundação Oswaldo Cruz Instituto René Rachou Núcleo de Estudos em Saúde Pública e Envelhecimento Belo Horizonte MG Brazil Fundação Oswaldo Cruz. Instituto René Rachou. Núcleo de Estudos em Saúde Pública e Envelhecimento. Belo Horizonte, MG, Brazil; III Secretaria Municipal de Saúde de Belo Horizonte Belo Horizonte MG Brazil Secretaria Municipal de Saúde de Belo Horizonte. Belo Horizonte, MG, Brazil

**Keywords:** Elderly Health, Health Policy, Qualitative Research

## Abstract

**OBJECTIVE:**

To understand the perception of different actors involved in the older adults care process in the intersectoral strategy of the *Programa Maior Cuidado* (PMC – Greater Care Program), aiming at the development of actions that contribute to the improvement of the services provided.

**METHODS:**

Eleven qualitative interviews guided by a semi-structured script were conducted in 2020 with key informants directly involved in the PMC: the older adults and their families, caregivers, health professionals and social assistance. In addition, to understand the functioning and proposals of the PMC, a documentary analysis was also carried out with the tracking of existing information on the guidelines, protocols, and management instruments. The content analysis technique was used to classify textual data, and the interpretation process was mediated by the theoretical-methodological framework of hermeneutic anthropology.

**RESULTS:**

Two categories were identified: “Repercussions of the care offered by the PMC: the ‘little’ that makes a difference” and “Problems beyond the PMC: the limits of family care in the face of violence against the older adults”. For all interviewees, the perception the PMC is very necessary is unison, being able to minimize the occurrence of health problems and avoid transfers of the older adults to hospitals and Long Stay Institutions for the Elderly (*Instituição de Longa Permanência* - ILPI in Portuguese). Chronic comorbidities increase the demands of health care and generate situations that can be managed by the PMC caregiver. Population aging requires the planning of strategies and public policies aimed at providing continuous care for the older adults, including those living in communities. The PMC emerges as an intersectoral alternative to assist in this issue.

**CONCLUSIONS:**

The PMC can be considered a good practice model to be expanded to other locations, however there are gaps that need to be rediscussed so that its processes are improved and its results enhanced.

## INTRODUCTION

Population aging introduces economic, social protection, and improvement issues in health care for the older adults^1^, in addition to inaugurating discussions about the need for health policies aimed at providing care to dependent older adults^2^.

In parallel to demographic and socioeconomic issues, the provision of care to the older adults reflects multivariate perceptions about old age, family rearrangements and the greater role of women – the main caregiver – in addition to domestic tasks^3^. By extension, older adults can also have their life condition defined from the care offered to them^2^, favoring their stay at home and avoiding hospitalizations and institutionalizations, costly outcomes for the public system and society^4^. Even for independent individuals to perform activities of daily living the presence of close people is reflected in well-being and quality of life^3^.

In Brazil, since 2008, there is the *Program Acompanhante do Idoso* (PAI – Companion of the Older Adult), in the city of São Paulo (SP). There are 49 multidisciplinary teams, each of them working in a Basic Health Unit, with coordinator, physician, nurse, two nursing assistants/technicians, administrative assistant and ten older adult companions, to guide the provision of home care to about 5,800 older adults in fragile situations^5^. In Belo Horizonte (MG), in 2011, the *Programa Maior Cuidado* (PMC – Greater Care Program) was instituted, an intersectoral policy – co-management between the municipal secretariats of Health and Social Care – for home care for dependent and semi-dependent older adults living in conditions of clinical and social vulnerability. With simpler design, at the local level, the PMC is performed in all 34 Reference Centers in Social Assistance (*Centro de Referência em Assistência Social* – CRAS in Portuguese) and half (72) of the teams of Health Centers^6^. The PMC offers a “social caregiver” – a professional trained for home care for the older adults – who works on days and times previously established by a multidisciplinary team, according to the degree of dependence and complexity of each case. The hiring of caregivers is carried out by a Civil Society Organization affiliated to the municipality. The care routine for the hired professionals includes support for personal hygiene, food, medication, guided physical exercises, diaper changes and dressings, as well as recreational and social activities that promote the participation of the older adults. Among the eligibility criteria for admission to the PMC, we highlight age (equal to or greater than 60 years); the classification of the degree of functionality – dependent or semi-dependent – for the performance of activities of daily living and socioeconomic evaluation that confirms vulnerability. Currently, 167 social caregivers monthly assist about 650 families of the older adults within this and/or other support networks. The PMC is fully funded by the Municipal Treasury, without co-financing of federal policies. The objective of this article was to understand, with the help of the qualitative approach, the perception of actors involved in the older adults care process about the functioning of this intersectoral public program.

## METHODS

Data presented are based on the qualitative branch of a larger study, which used a multi-method approach^7^, proposed by the René Rachou Institute (Fiocruz/MG) and approved by the institution’s ethics committee (CAAE: 96033418.9.0000.5091). This is an international partnership signed between the Medical Research Council (UK), Fundação de Amparo à Pesquisa do Estado de Minas Gerais and Fundação Cearense de Apoio ao Desenvolvimento Científico e Tecnológico, whose basic objective is to support public policies from the identification of successful practices that contribute to the reduction of unnecessary and prolonged admissions and hospitalizations of Brazilian older adults in hospitals and *Instituições de Longa Permanência para Idosos* (ILPI).

Studies with qualitative components can help understanding the functioning of services based on the experience lived by the actors involved – professionals and users^8^, the identification of potentialities, and possible bottlenecks faced in practice.

In 2020, 11 interviews were conducted with a semi-structured script together with key informants, being: four older adults (and, in the same interview, their respective family caregivers); two technical references of the PMC representatives of the Health and Social Assistance axes; two professionals of the Health Center and three caregivers of the Program (social caregiver – PMC). The final number of interviewees was regulated and determined by the criterion of empirical data saturation^9^. Due to the frailty of the older adults participants in this study (memory difficulties, an older adult woman bedridden in an advanced stage of Alzheimer’s, etc.), the reports of family caregivers were more prevalent compared to those of the assisted older adult.

All interviews were recorded and later transcribed literally. In addition, to understand the functioning and proposals of the PMC, existing information on the guidelines, protocols, and management instruments were analyzed.

Data were systematized using the Bardin content analysis technique^10^, performed in three stages:

- Pre-analysis, in which each interview was transcribed and identified to facilitate the organization of the data;
- Exploration of the material, through fluctuating readings in all responses of each interviewee for the definition of categories of analysis; and also
- The interpretation of the results after reflective analysis^10^, based on a careful and critical reading of the speeches, with the definition of codes to form categories of incidence and contextual similarity.

The data were organized in spreadsheets in the Excel Program and all excerpts were categorized and coded, allowing the identification of themes and subjects of greater relevance. From this categorization, were performed: 1) critical reading of responses; 2) analytical reflection; and 3) identification of final categories. To ensure the analysis validation, classification was performed independently by three researchers. The entire analysis process was emic and guided by the theoretical-methodological framework of hermeneutic anthropology, whose analysis is anchored to the interpretation of the meaning that social groups attribute to certain practices, considering the sociocultural context of action and the factors that influence it^11^.

To ensure anonymity, the participants were identified, respectively, according to the category belonging to the PMC – older adult user, family caregiver, social caregiver, Technical Reference (RT) of the PMC health and/or Social Assistance axis, gender (F for female and M for male) and their age.

## RESULTS

Regarding the characterization of the interviewees, two older adults were male and two were female, between 77 and 92 years old, and mean stay in the Program was 5.7 years. All three social caregivers were women between 25 and 44 years old, and had been working in the PMC for an average of 13 months. The four RT were women – three nurses and a social worker – with ages ranging from 43 to 60 years. The two family caregivers interviewed, one 42 years old and the other 61 years old, were daughters of the older adults.

The content analysis identified two major categories: “ *Repercussions of the care offered by the PMC: the ‘little’ that makes* a difference” and “ *Problems beyond the PMC: the limits of family care in the face of violence against the older adults*”, each of them organized into subcategories as will be presented below.

### Repercussions of Care Offered by PMC: The “Little” That Makes a Difference

In this category, the elements linked to the care offered by the PMC at home and its impact on the various actors involved are presented. From it, three subcategories emerged, as presented below.

In subcategory 1 – *Repercussions for the older adults* – the interlocutors identify the effectiveness of the PMC in responding to conditions of social vulnerability and health of the older adults:

*She [social caregiver] is here every day, looks at me, talks to me*… *helps me shower (*…*) If I feel dizzy it’s good to have someone to hold on to. (*…*). This program serves me well. There wasn’t even one that didn’t take good care of me. They all take care of me with the “greatest care” (Older adult 2. M. 92 years old).*

*In the short time they are in residence, we noticed the older adults had improvement in conditions or some did not worsen, which is already a great gain (*…*). This avoids not only hospitalization, but also (*…*) an early death due to lack of care ” (Nurse Health Center. F. 45 years old).*

*We noticed improvement, both in mood and in coexistence. There are older adults who come to the house and they do not walk, because they have some difficulty. Then we start doing some activities, with the guidance of the physical therapist, and then they become more active and we see an improvement. Because let’s think like this: the physical therapist gives some guidance there at the Post, but if there is no one to help you, it’s no use. (*…*) more often than not, the family member does not have time for these issues (Social caregiver PMC. F. 44 years old).*

In the universe researched, the role of caregivers at home is emphasized, albeit for *a short time,* as small actions that make a lot of difference, especially in the recovery of functionality of the older adults. In addition, the interlocutors consider that the health of the family nucleus is affected by the need for daily care, as explained in subcategory 2 – *Repercussions for the family*:

*The dependent or semi-dependent older adult makes an entire family sick (*…*).*

*(RT PMC – Health. F. 43 years old).*

*My mother was already gone [deceased] without the caregiver... because I had some phases of getting very tired, exhausted from having to take care of my mother (Family caregiver – daughter. F. 61 years old).*

*I, as a caregiver, working in that house, at that time when the family member needs to leave, he will leave and return quietly and safely, for having left the older adult with someone (Social caregiver PMC. F. 44 years old).*

In addition to observing the positive impact on reducing the burden of care imposed on the family member, the PMC functions as a source of income and a tool of social inclusion for the social caregivers themselves, as presented in subcategory 3 – *Repercussions for the social caregiver*:

*It is a program that also helps caregivers. Also residents of areas with high social vulnerability, violence etc. This also influences their sensitivity with care for the older adults and also the pride of “wearing the shirt” of the Program. (*…*) Some had long been unemployed and found this job opportunity. In one of the trainings that the central level does with these caregivers, (*…*) the facilitator asked them to take an object that referred to the meaning of the Program in their lives (*…*) several caregivers took the work card with the registration of the contract (RT PMC – Health. F. 43 years old).*

Convinced that the Program avoids physical and mental problems, prevents early deaths, helps in functional recovery, allows a breath for family members and translates into care for the person assisted, the different actors also recognize their limits, as will be seen in the next analytical category.

### Problems Beyond the PMC: The Limits of Family Care in the Face of Violence Against the Older Adults

This category presents several factors and conditions that cross the performance of the Program, including the presentation of vulnerabilities, difficulties and insufficiencies that the PMC faces, organized into three subcategories.

Subcategory 1 – Poverty, vulnerability, food insecurity:

*The program itself is very good, but it has some problems that go beyond that. How can we solve the problems of poverty and vulnerability? It’s something that is a limit for us. (*…*) The Program comes to help the older adults, unburden the family, go out with the older adults, give a bath. But sometimes we come to the house and have to use our imagination to try to make something for him to eat. Or what’s worse: sometimes there’s absolutely nothing to eat (Social caregiver PMC. F. 31 years old).*

*We have many precarious conditions of hygiene, of food. So, it is not enough just to be careful with techniques. There really is a need for economic care, food, a decent place to live. Without the basics, it ends up aggravating a situation that could be minimally avoided if the family had any condition (Nurse Health Center. F. 45 years old).*

Many of the low-income families assisted are unable to provide basic care, adequate food and sanitary conditions, or to hire caregivers or people to assist them in caring for the older adults.

Subcategory 2 – Insufficiency of care linked to violation of rights:

*When the caregiver reports to us, with all care, that there is abandonment (*…*): “I have been trying to make tea for a week, but there is no tea. B-U-T”. She has already asked the family to buy or that the family has the benefit card of the older adults and the food is missing. These are situations that go beyond the performance and attributions of the caregiver” (RT PMC – Social Assistance. F. 48 years old).*

*There is a family that recognizes the caregiver more as an intruder inside the house, especially when it begins to realize that the caregiver acts as “eyes and ears”, and ends up no longer wanting this person inside the house” (RT PMC – Saúde. F. 43 years old).*

Abandonment or neglect of the family reveals the insufficiency of care linked to the risk or violation of rights and, often, the caregivers of the PMC are spokespersons for situations that involve a myriad of complexities beyond professional performance.

Subcategory 3 – Institutionalization as a last option:

*When we’ve exhausted everything we could do. For example, changing caregivers at home. In some cases, we perceive a situation of violation with an accountability of the people who live in that house (RT PMC – Social Assistance. F. 48 years old).*

*When all possibilities of attempts to improve the bond of the older adults with the family are exhausted. Because*… *it’s not always due to negligence (*…*). It is the reflection of a construction well done or not, throughout the life story of that family. (*…*) We also find it very difficult [to institutionalize], especially with vacancies. And even more, to prove that this situation is happening (RT PMC – Health. F. 43 years old).*

One of the basic objectives of the PMC is to avoid and/or delay institutionalizations. However, there are issues of social vulnerability, violation of rights, and family dynamics that make the continuity of care unfeasible. In these cases, institutionalization appears as the last/only option.

In Figure, a schematic synthesis of the Program’s performance as a model of good practice is presented, based on the perception of the interviewed actors and the documentary analysis carried out.

**Figure:**
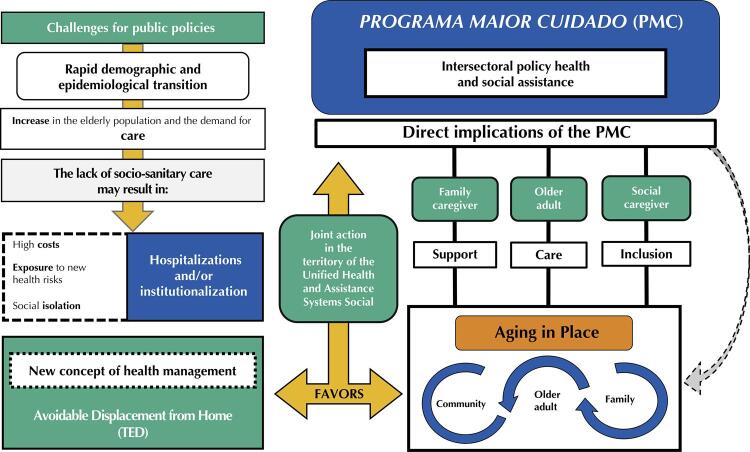
Summary of the implications of the Programa Maior Cuidado as a model of good practice.

Source: analytical synthesis elaborated by the authors from the empirical data collected.

## DISCUSSION

As seen in Figure, demographic and epidemiological transitions occur with greater demand for chronic care due to exacerbations, worsening or complications of Non-communicable Chronic Diseases^12^, which are mainly responsible for hospital admissions and clinical emergencies for the Brazilian older adults^13^. Thus, it is crucial to create new strategies or restructure existing health services to prevent potential diseases that may affect the older adults population. Absent or insufficient socio-sanitary care can lead to overload of health services and, by cascading effect, result in hospitalizations and institutionalizations^14^.

When analyzing the predictors of hospital admission and suggesting the new concept of Avoidable Displacement from Home (*Transferência Evitável de Domicílio* - TED in Portuguese), Lloyd-Sherlock et al.^15^ propose that policymakers define criteria to characterize unnecessary/prolonged hospital admissions and institutionalizations of the older adults and improve existing protocols and practices^15^. The PMC is able to minimize or avoid the number of TEDs by offering direct home care, which, by extension, enables a form of surveillance – based on adequate guidance and control – for older adults with high potential for clinical diseases. A quantitative study on the use of health services among PMC users showed that the assisted population has more access to rehabilitation and planned consultations in the health network than the unattended^16^. From this perspective, the PMC can be classified as a model of good practice, to be expanded to other Brazilian states and municipalities, in addition to being understood as a social innovation, a care technology that contributes to the prevention and treatment of this public in its own habitat, that is, the Aging in Place (AiP). AiP means having the health and social support needed to live safely at home or in the community in the aging process^17^.

In addition, the greater the frailty and functional dependence, in situations of extreme social vulnerability, such as the population assisted by the PMC, new forms of approach to care are necessary^18^. “Minimal” care, such as counseling about a medication, can generate positive and significant repercussions for the older adult, their family and the health system itself, by reducing the chances of clinical complications^19^.

When discussing “*the little that makes a difference*”, reported and/or implied throughout the interviews, it is revealed that those who practice and live the Program recognize its importance; however, the notion of care as “little” may reflect the understanding of care as something natural, minor, anchored in the certainty that someone, usually a woman, will do it^20^. The actors involved in the PMC seem unaware that they participate in one of the rare public policy offerings of continuing care exclusively for the older adults in low-income countries, as already recognized by the World Health Organization (WHO)^21^.

Usually, the family caregiver does not have the preparation or technical skills necessary for the care of an older adult^22^. In this study, family caregivers were daughters of the older adult and reported work overload and abandonment of their own activities in favor of the care of the other. Women constitute the majority of the older adult population, spend more time exposed to pathophysiological risks and are the main caregivers^22^. As vulnerable to care issues, they deserve the attention of managers and health systems.

In matters related to the family, the PMC contributes to family caregivers in practical day-to-day work, helping them to resume their daily activities and reducing part of the work overload imposed by care. When the family does not present conditions (psychological, financial, social and even human) for care, the older adult is exposed to risky situations^23^. There is talk of family insufficiency, a condition that compromises the functionality and quality of life of the older adult. However, this is not a matter of family insufficiency, but of insufficient care policies to support families in their need for care^23^.

In this context, once the possibilities of solving problems have been exhausted, institutionalization is considered the best care option. However, bottlenecks are identified in the public network to effect this form of care. A study carried out in 2014 identified that in Belo Horizonte, 73.1% of philanthropic ILPI had waiting lists, with 9.7% accepting only independent older adults; 20.1% only independent or semi-dependent older adults; 48.1% of ILPI did not receive older adults with dementia and 52.2% refused new residents who presented certain diseases, such as infectious ones^24^.

Thus, care is assumed as a transversal dimension of the health and well-being of citizens of all ages, and it is up to the State and society to seek equity in resources, the sharing of tasks between genders, and dignity in the care of people of all ages^23^.

A key point of the PMC is its intersectoriality that connects different actors and multidisciplinary teams and permeates all operations of the Program: joint meetings for case review, combined contributions in individualized care plans and continuous communication with caregivers^6^. Thus, the Program is part of the care networks, especially in primary care – a priority level to assist and monitor the health status of the older adults population^25^.

Another important element related to the role of social caregivers in the Program is their social inclusion through formal work. With salary comes the definition of specific roles and clear responsibilities, including working time in each household. Experiences with volunteer caregivers in countries such as Costa Rica and Thailand show that while these provide some support, their contributions are limited and inconsistent^26^.

The Program operates in communities that face problems and deprivations of the most diverse orders, but a central issue of the PMC is not to be restricted to the physical-functional health of each older adult, but to consider the situational context to offer more comprehensive strategies of support, carry out direct monitoring of the assisted older adult and reach the other people and policies involved, beyond the home. In the universe researched, for all different actors the perception the Program is very important is unified and presents strategies and positive implications for different axes of action. Its objectives and actions are clear, however, gaps persist to be rediscussed in the performance scenarios so that the Program has increasingly successful processes and results.

## CONCLUSION

This study carried out a qualitative analysis of the PMC – intersectoral action strategy that offers care to the older adults in situations of high clinical and social vulnerability in Belo Horizonte. The Program, as a social innovation, provides the opportunity for participants to develop a dignified old age, with support in activities and relief for the work overload of family caregivers. With actions and basic care guidelines, it proved to be an initiative capable of minimizing the occurrence of health problems, so to avoid hospitalizations and institutionalizations.

This article has as its main limitation the difficulties encountered during data collection due to the lack of registration and systematizations in regional health that adhered to the PMC, a situation that has been corrected from the research itself.

In this context of aging, listening to the actors involved in the PMC enabled an expanded understanding of the multiple issues involved in care and public policies, especially in areas of greater social vulnerability, such as the PMC’s locus of action. This research is expected to raise new discussions and strategies that contribute to the strengthening of the Program and construction of care policies for the older adults.
