# Supplementary material for: Greater Care Program in the face of the challenges of aging: a qualitative analysis
Source: Rev Saude Publica. 2023 Sep 29;57:70. doi: 10.11606/s1518-8787.2023057004859 (PMC10547383; doi:10.11606/s1518-8787.2023057004859)
Supplement: Translation

# Translation: Programa Maior Cuidado diante dos desafios do envelhecimento: uma análise qualitativa

**Authors:** Cláudio Phillipe Fernandes de Castro, Janaína de Souza Aredes, Karla Cristina Giacomin, Josélia Oliveira Araújo Firmo

**Affiliations:** I Fundação Oswaldo Cruz Instituto René Rachou Programa de Pós-graduação em Saúde Coletiva Belo Horizonte MG Brasil Fundação Oswaldo Cruz. Instituto René Rachou. Programa de Pós-graduação em Saúde Coletiva. Belo Horizonte, MG, Brasil; II Fundação Oswaldo Cruz Instituto René Rachou Núcleo de Estudos em Saúde Pública e Envelhecimento Belo Horizonte MG Brasil Fundação Oswaldo Cruz. Instituto René Rachou. Núcleo de Estudos em Saúde Pública e Envelhecimento. Belo Horizonte, MG, Brasil; III Secretaria Municipal de Saúde de Belo Horizonte Belo Horizonte MG Brasil Secretaria Municipal de Saúde de Belo Horizonte. Belo Horizonte, MG, Brasil

**Keywords:** Saúde do Idoso, Política de Saúde, Pesquisa Qualitativa

## Abstract

**OBJETIVO:**

Compreender a percepção de diferentes atores envolvidos no processo de cuidado ao idoso na estratégia intersetorial do Programa Maior Cuidado (PMC), visando o desenvolvimento de ações que contribuam para a melhoria dos serviços prestados.

**MÉTODOS:**

Foram realizadas 11 entrevistas qualitativas guiadas por roteiro semiestruturado em 2020 junto a informantes-chave diretamente envolvidos no PMC: idosos e seus familiares, cuidadores, profissionais da saúde e da assistência social. Adicionalmente, com o objetivo de compreensão do funcionamento e das propostas do PMC, também foi realizada uma análise documental com o rastreamento de informações existentes sobre as diretrizes, os protocolos e os instrumentos de gestão. Utilizou-se a técnica de análise de conteúdo para classificar os dados textuais, e o processo de interpretação foi mediado pelo referencial teórico-metodológico da antropologia hermenêutica.

**RESULTADOS:**

Foram identificadas duas categorias: “Repercussões do cuidado ofertado pelo PMC: o ‘pouco’ que faz diferença” e “Problemas para além do PMC: os limites do cuidado familiar diante da violência contra a pessoa idosa”. Para todos os entrevistados é uníssona a percepção de que o PMC é muito necessário, sendo capaz de minimizar a ocorrência de agravos de saúde e evitar transferências dos idosos para hospitais e Instituições de Longa Permanência para Idosos (Ilpi). As comorbidades crônicas aumentam as demandas de cuidado em saúde e geram situações que podem ser manejadas pelo cuidador do PMC. O envelhecimento populacional requer o planejamento de estratégias e políticas públicas voltadas para o provimento de cuidados contínuos para idosos, incluindo aqueles que vivem em comunidades. O PMC surge como alternativa intersetorial para auxiliar nessa questão.

**CONCLUSÕES:**

O PMC pode ser considerado um modelo de boa prática a ser expandido para outras localidades, entretanto existem lacunas que necessitam ser rediscutidas para que seus processos sejam aprimorados e seus resultados potencializados.

## INTRODUÇÃO

O envelhecimento populacional introduz questões econômicas, de proteção social e de melhorias na atenção à saúde da pessoa idosa^1^, além de inaugurar discussões acerca da necessidade de políticas de saúde voltadas ao provimento de cuidados a pessoas idosas dependentes^2^.

Em paralelo às questões demográficas e socioeconômicas, a oferta do cuidado à pessoa idosa repercute percepções multivariadas sobre a velhice, rearranjos familiares e o maior protagonismo da mulher – principal cuidadora – para além das tarefas domésticas^3^. Por extensão, as pessoas idosas também podem ter sua condição de vida definida a partir do cuidado que lhes é ofertado^2^, favorecendo a permanência delas no domicílio e evitando hospitalizações e institucionalizações, desfechos onerosos para o sistema público e à sociedade^4^. E, mesmo para indivíduos independentes realizarem as atividades de vida diária, a presença de pessoas próximas reflete-se em bem-estar e qualidade de vida^3^.

No Brasil, desde 2008, existe o Programa Acompanhante do idoso (PAI), na cidade de São Paulo (SP). São 49 equipes multidisciplinares, cada uma delas lotada em uma unidade básica de saúde, contando com coordenador, médico, enfermeiro, dois auxiliares/técnicos de enfermagem, auxiliar administrativo e dez acompanhantes de idosos, para orientar a prestação de cuidados domiciliares a cerca de 5.800 idosos em situação de fragilidade^5^. Em Belo Horizonte (MG), no ano de 2011, foi instituído o Programa Maior Cuidado (PMC), uma política intersetorial – cogestão entre as Secretarias Municipais de Saúde e Assistência Social – para o cuidado domiciliar a idosos dependentes e semidependentes que vivem em condições de vulnerabilidade clínica e social. De desenho mais simples, em nível local, o PMC é executado em todos os 34 Centros de Referência em Assistência Social (Cras) e metade (72) das equipes dos centros de saúde^6^. O PMC oferece um “cuidador social” – profissional capacitado para o cuidado domiciliar a idosos –, que trabalha em dias e horários previamente estabelecidos por equipe multidisciplinar, conforme o grau de dependência e a complexidade de cada caso. A contratação dos cuidadores é realizada por uma Organização da Sociedade Civil conveniada ao município. A rotina de cuidado para os profissionais contratados inclui apoio para higiene pessoal, alimentação, medicação, exercícios físicos orientados, trocas de fraldas e curativos, além de atividades recreativas e sociais que promovam a participação do idoso. Dentre os critérios de elegibilidade para ingresso no PMC destacam-se a idade (igual ou superior a 60 anos); a classificação do grau de funcionalidade – dependente ou semidependente – para a realização de atividades de vida diária e avaliação socioeconômica que confirme a vulnerabilidade. Atualmente, 167 cuidadores sociais assistem, mensalmente, cerca de 650 famílias dos idosos inseridos e/ou outras redes de apoio. O PMC é totalmente custeado pelo Tesouro Municipal, sem cofinanciamento de políticas federais. O objetivo deste artigo foi compreender, com a ajuda da abordagem qualitativa, a percepção de atores envolvidos no processo de cuidado ao idoso acerca do funcionamento desse programa público intersetorial.

## MÉTODOS

Os dados apresentados baseiam-se no braço qualitativo de um estudo maior, que utilizou uma abordagem multimétodo^7^, proposto pelo Instituto René Rachou (Fiocruz/MG) e aprovado pelo comitê de ética da instituição (CAAE: 96033418.9.0000.5091). Trata-se de uma parceria internacional firmada entre o Medical Research Council (Reino Unido), Fundação de Amparo à Pesquisa do Estado de Minas Gerais e Fundação Cearense de Apoio ao Desenvolvimento Científico e Tecnológico , cujo objetivo basilar é apoiar políticas públicas, a partir da identificação de práticas exitosas que contribuam para a redução de admissões e internações desnecessárias e prolongadas de idosos brasileiros em hospitais e Instituições de Longa Permanência para Idosos (Ilpi).

Estudos com componentes qualitativos podem ajudar a entender o funcionamento dos serviços a partir da experiência vivida pelos próprios atores envolvidos – profissionais e usuários^8^, da identificação de potencialidades e possíveis gargalos enfrentados na prática.

Em 2020, foram realizadas 11 entrevistas guiadas por roteiro semiestruturado, junto a informantes-chave, sendo: quatro idosos (e, na mesma entrevista, suas respectivas cuidadoras familiares); duas referências técnicas do PMC representantes dos eixos Saúde e Assistência Social; dois profissionais do Centro de Saúde e três cuidadores do Programa (cuidador social – PMC). O número final de entrevistados foi regulado e determinado pelo critério de saturação empírica dos dados^9^. Devido à fragilidade dos idosos participantes deste estudo (dificuldades de memória, uma idosa acamada em estágio avançado de Alzheimer etc.), os relatos dos cuidadores familiares tiveram maior preponderância, se comparados àqueles da pessoa idosa assistida.

Todas as entrevistas foram gravadas e, posteriormente, transcritas de modo literal. Adicionalmente, visando compreender o funcionamento e as propostas do PMC, foram analisadas informações existentes sobre as diretrizes, os protocolos e os instrumentos de gestão.

Os dados foram sistematizados a partir da técnica de análise de conteúdo de Bardin^10^, realizada em três etapas:

- Pré-análise, em que cada entrevista foi transcrita e identificada para facilitar a organização dos dados;
- Exploração do material, por meio de leituras flutuantes em todas as respostas de cada entrevistado para a definição de categorias de análise; e ainda
- A interpretação dos resultados após a análise reflexiva^10^, a partir de uma leitura atenta e crítica das falas, com definição de códigos para formar categorias de incidência e semelhança contextual.

Os dados foram organizados em planilhas no Programa Excel e todos os excertos categorizados e codificados, permitindo identificar temas e assuntos de maior relevância. A partir dessa categorização aconteceram: 1) leitura crítica das respostas; 2) reflexão analítica; e 3) identificação de categorias finais. Para garantir a validação da análise, a classificação foi realizada de modo independente por três pesquisadores. Todo o processo de análise foi êmico e orientado pelo referencial teórico-metodológico da antropologia hermenêutica, cuja análise ancora-se à interpretação do significado que os grupos sociais atribuem a determinadas práticas, considerando-se o contexto sociocultural de ação e os fatores que o influenciam^11^.

Para assegurar o anonimato, os participantes foram identificados, respectivamente, conforme a categoria pertencente no PMC – usuário idoso, cuidador familiar, cuidador social, Referência Técnica (RT) do PMC eixo Saúde e/ou Assistência Social, sexo (F para feminino e M para masculino) e a respectiva idade.

## RESULTADOS

Quanto à caracterização dos entrevistados, dois idosos eram do sexo masculino e dois do sexo feminino, com idades entre 77 e 92 anos e média de permanência no Programa de 5,7 anos. Todas as três cuidadoras sociais eram mulheres, com idade entre 25 e 44 anos e trabalhavam no PMC em média há 13 meses. As quatro RT eram mulheres – três enfermeiras e uma assistente social –, com idade entre 43 e 60 anos. As duas cuidadoras familiares entrevistadas, uma de 42 anos e outra de 61 anos, eram filhas dos idosos.

À análise de conteúdo foram identificadas duas categorias maiores: “*Repercussões do cuidado ofertado pelo PMC: o ‘pouco’ que faz diferença*” e “*Problemas para além do PMC: os limites do cuidado familiar diante da violência contra a pessoa idosa*”, cada uma delas organizadas em subcategorias como será apresentado a seguir.

### Repercussões do Cuidado Ofertado pelo PMC: o “Pouco” que Faz Diferença

Nesta categoria, são apresentados os elementos vinculados ao cuidado ofertado pelo PMC no domicílio e seu impacto junto aos diversos atores envolvidos. Dela, emergiram três subcategorias apresentadas a seguir.

Na subcategoria 1 – *Repercussões para a pessoa idosa* – os interlocutores identificam a efetividade do PMC em responder a condições de vulnerabilidade social e de saúde dos idosos:

*Ela [cuidadora social] está aqui todo dia, me olha, conversa comigo*… *me ajuda a tomar banho (*…*) Se eu tiver uma tontura é bom ter alguém para segurar. (*…*). Esse Programa me atende até muito bem. Não tem uma que não cuidou bem de mim. Todas cuidam de mim com o “maior cuidado” (Idoso 2. M. 92 anos).*

*No pouco tempo que eles estão na residência, nós notamos que os idosos tiveram melhora no quadro ou alguns não agravaram, o que já é um grande ganho (*…*). Isso evita não só internação, mas também (*…*) um óbito precoce por falta de cuidado “ (Enfermeira centro de saúde. F. 45 anos).*

*Notamos melhora, tanto no humor quanto na convivência. Tem idosos que a gente chega na casa e eles não andam, por terem alguma dificuldade. Aí a gente começa a fazer algumas atividades, com a orientação da fisioterapeuta e daí eles ficam mais ativos e a gente vê uma melhora. Porque vamos pensar assim: a fisioterapeuta orienta lá no Posto, mas se não tem quem o ajude, não adianta. (*…*) na maioria das vezes, o familiar não tem tempo para essas questões (Cuidador social PMC. F. 44 anos).*

No universo pesquisado, enfatiza-se a atuação dos cuidadores no domicílio, ainda que por *pouco tempo,* enquanto pequenas ações que fazem muita diferença, sobretudo na recuperação da funcionalidade do idoso. De forma complementar, os interlocutores consideram que a saúde do núcleo familiar é afetada pela necessidade de cuidado diário, como exposto na subcategoria 2 – *Repercussões para a família*:

*O idoso dependente ou semidependente adoece uma família inteira (*…*).*

*(RT PMC – Saúde. F. 43 anos).*

*A minha mãe já tinha ido [falecido] sem o cuidador*… *porque eu tive umas fases de ficar muito cansada, esgotada por ter que cuidar da minha mãe (Cuidadora familiar – Filha. F. 61 anos).*

*Eu, como cuidadora, atuando naquela casa, naquele horário que o familiar precisa sair, ele vai sair e voltar tranquilo e com segurança, por ter deixado o idoso com alguém (Cuidador social PMC. F. 44 anos).*

Além de observar o impacto positivo na redução da sobrecarga do cuidado imposta ao familiar, o PMC funciona como fonte de renda e ferramenta de inclusão social para os próprios cuidadores sociais, como apresentado na subcategoria 3 – *Repercussões para o cuidador social*:

*É um Programa que também ajuda os profissionais cuidadores. Também moradores de áreas com alta vulnerabilidade social, violência etc. Isso também influencia nessa sensibilidade deles com o cuidado ao idoso e também ao orgulho de “vestir a camisa” do Programa. (*…*) Alguns estavam há muito tempo desempregados e encontraram essa oportunidade de trabalho. Em um dos treinamentos que o nível central faz com esses cuidadores, (*…*) a facilitadora pediu para que eles levassem um objeto que remetesse ao significado do Programa na vida deles (*…*) vários cuidadores levaram a carteira de trabalho com o registro do contrato (RT PMC – Saúde. F. 43 anos).*

Convencidos de que o Programa evita agravos físicos e mentais, previne óbitos precoces, ajuda na recuperação funcional, possibilita um respiro para os familiares e se traduz em cuidado para a pessoa assistida, os diferentes atores também reconhecem os seus limites, como será visto na próxima categoria analítica.

### Problemas para Além do PMC: os Limites do Cuidado Familiar diante da Violência contra a Pessoa Idosa

Essa categoria apresenta diversos fatores e condições que atravessam a atuação do Programa, incluindo a apresentação das vulnerabilidades, dificuldades e insuficiências que o PMC enfrenta, organizadas em três subcategorias.

Subcategoria 1 – Pobreza, vulnerabilidade, insegurança alimentar:

*O Programa em si é muito bom, mas tem alguns problemas que vão além. Como resolver as questões de pobreza, da vulnerabilidade? É algo que temos o nosso limite. (*…*) O Programa vem para ajudar o idoso, desafogar a família, sair com o idoso, dar um banho. Mas, às vezes, a gente chega na casa e tem que usar a imaginação para tentar fazer alguma coisa para ele comer. Ou o que é pior: às vezes não tem absolutamente nada para comer (Cuidador social PMC. F. 31 anos).*

*Temos muitas condições precárias de higiene, de alimentação. Então, não basta somente o cuidado com técnicas. Precisa realmente do cuidado econômico, de comida, de lugar decente para viver (*…*). Sem o básico acaba agravando um quadro que poderia ser minimamente evitado se a família tivesse alguma condição (Enfermeira centro de saúde. F. 45 anos).*

Muitas das famílias de baixa renda assistidas não têm condições de oferecer cuidados básicos, alimentos e condições sanitárias adequadas, nem de contratar cuidadores ou pessoas que as auxiliem no cuidado ao idoso.

Subcategoria 2 – Insuficiência de cuidados ligada à violação de direitos:

*Quando a cuidadora nos reporta, com todo o cuidado, que há abandono (*…*): “tem uma semana que eu estou tentando fazer um chá, só que não tem nenhum chá. S-Ó Q-U-E”. Ela já pediu para que a família comprasse ou que a família está com o cartão do idoso do benefício e a comida falta. São situações que vão além da atuação e das atribuições da cuidadora” (RT PMC – Assistência Social. F. 48 anos).*

*Tem família que reconhece o cuidador mais como um intruso dentro da casa, principalmente quando começa a perceber que o cuidador são “olhos e ouvidos”, acaba não querendo mais essa pessoa dentro de casa” (RT PMC – Saúde. F. 43 anos).*

Abandono ou negligência familiar revelam a insuficiência de cuidados ligada ao risco ou à violação de direitos e, muitas vezes, os cuidadores do PMC são porta-vozes de situações que envolvem uma miríade de complexidades para além da atuação profissional.

Subcategoria 3 – Institucionalização como última opção:

*Quando a gente já esgotou tudo aquilo que a gente poderia fazer. Como por exemplo, troca de cuidador na casa. Em alguns casos, a gente percebe uma situação de violação com uma responsabilização das pessoas que vivem naquela casa (RT PMC – Assistência Social. F. 48 anos).*

*Quando esgotam todas as possibilidades de tentativas de melhorar o vínculo do idoso com a família. Porque*… *nem sempre é por negligência (*…*). É o reflexo de uma construção bem-feita ou não, ao longo da história de vida daquela família. (*…*) A gente também encontra muita dificuldade [para institucionalizar], especialmente com vagas. E mais ainda, provar que essa situação está acontecendo (RT PMC – Saúde. F. 43 anos).*

Um dos objetivos basilares do PMC é evitar e/ou retardar as institucionalizações. Porém, há questões de vulnerabilidade social, de violação de direitos e da dinâmica familiar que inviabilizam a continuidade do cuidado. Nesses casos, a institucionalização aparece como última/única opção.

Na Figura, é apresentada uma síntese esquemática da atuação do Programa enquanto um modelo de boa prática, a partir da percepção dos atores entrevistados e da análise documental realizada.

**Figura:** Síntese sobre as implicações do Programa Maior Cuidado enquanto um modelo de boa prática.

Fonte: síntese analítica elaborada pelos autores a partir dos dados empíricos coletados.

## DISCUSSÃO

Como visto na Figura, as transições demográfica e epidemiológica cursam com maior demanda por cuidados crônicos decorrente com exacerbações, piora ou complicações das Doenças Crônicas Não Transmissíveis^12^, principais responsáveis por internações hospitalares e emergências clínicas para idosos brasileiros^13^. Assim, revela-se crucial criar novas estratégias ou reestruturar serviços de saúde existentes para prevenir potenciais agravos que podem atingir a população idosa. Cuidados sociossanitários ausentes ou insuficientes podem levar à sobrecarga dos serviços de saúde e, por efeito cascata, resultar em hospitalizações e institucionalizações^14^.

Ao analisar os preditores de admissão hospitalar e sugerir o novo conceito de Transferência Evitável do Domicílio (TED), Lloyd-Sherlock et al.^15^ propõem que os formuladores de políticas definam critérios para caracterizar admissões desnecessárias/prolongadas em hospitais e institucionalizações de idosos e aprimorem os protocolos e as práticas existentes^15^. O PMC é capaz de minimizar ou evitar o número de TED por meio da oferta do cuidado direto no domicílio, o que, por extensão, viabiliza uma forma de vigilância – a partir de orientações e controle adequados – para idosos com alto potencial para agravos clínicos. Um estudo quantitativo sobre a utilização de serviços de saúde entre usuários do PMC evidenciou que a população assistida tem mais acesso à reabilitação e consultas planejadas na rede de saúde do que a não assistida^16^. Nessa perspectiva, o PMC pode ser classificado como um modelo de boa prática, a ser expandido para outros estados e municípios brasileiros, além de ser compreendido como uma inovação social, uma tecnologia de cuidado que contribui para prevenção e tratamento desse público no seu próprio habitat, isto é, o *Aging in Place* (AiP). O AiP significa ter saúde e apoio social necessários para viver com segurança em casa ou na comunidade no processo de envelhecimento^17^.

Ademais, quanto maior a fragilidade e a dependência funcional, em situação de extrema vulnerabilidade social, como a população assistida pelo PMC, novas formas de abordagem para o cuidado se fazem necessárias^18^. Um cuidado “mínimo”, como orientar sobre uma medicação, pode gerar repercussões positivas e significativas para a pessoa idosa, a sua família e para o próprio sistema de saúde, ao reduzir as chances de complicações clínicas^19^.

Ao discutir “*o pouco que faz diferença*”, relatado e/ou subentendido ao longo das entrevistas, revela-se que quem pratica e vive o Programa reconhece sua importância, contudo a noção de cuidado como “pouco” talvez reflita a compreensão do cuidado como algo natural, menor, ancorado na certeza de que alguém, geralmente uma mulher, o fará^20^. Os atores envolvidos no PMC parecem desconhecer que participam de uma das raras ofertas de política pública de cuidados continuados exclusivamente a idosos em países de baixa renda, conforme já reconhecido pela Organização Mundial da Saúde (OMS)^21^.

Usualmente, o cuidador familiar não dispõe de preparo ou habilidades técnicas necessárias para o cuidado a uma pessoa idosa^22^. Neste estudo, as cuidadoras familiares eram filhas dos idosos e relatavam a sobrecarga de trabalho e o abandono das suas próprias atividades em prol do cuidado do outro. As mulheres constituem a maior parte da população idosa, passam mais tempo expostas aos riscos fisiopatológicos e são as principais cuidadoras^22^. Como vulneráveis a questões do cuidado, elas merecem a atenção dos gestores e dos sistemas de saúde.

Nas questões relativas à família, o PMC contribui com os cuidadores familiares no trabalho prático do dia a dia, auxiliando-os a retomar suas atividades cotidianas e reduzindo parte da sobrecarga de trabalho imposta pelo cuidado. Quando a família não apresenta condições (psicológicas, financeiras, sociais e até mesmo humanas) de cuidar, a pessoa idosa fica exposta a situações de risco^23^. Fala-se de insuficiência familiar, condição que compromete a funcionalidade e a qualidade de vida dos idosos. Porém, não se trata de insuficiência familiar, mas sim da insuficiência de políticas de cuidados que apoiem as famílias na sua necessidade de cuidar^23^.

Nesse contexto, esgotadas as possibilidades de resoluções dos problemas, a institucionalização é considerada a melhor opção de cuidado. Contudo, gargalos são identificados na rede pública para efetivar esta forma de cuidado. Um estudo realizado em 2014, identificou que em Belo Horizonte, 73,1% das Ilpi filantrópicas apresentavam listas de espera, sendo que 9,7% aceitavam somente idosos independentes; 20,1% apenas idosos independentes ou semidependentes; 48,1% das Ilpi não recebiam idosos com demências e 52,2% recusavam novos residentes que apresentassem certas doenças, como as infecciosas^24^.

Assume-se, assim, o cuidado como uma dimensão transversal da saúde e do bem-estar dos cidadãos de todas as idades, cabendo ao Estado e à sociedade buscar pela equidade nos recursos, pela partilha das tarefas entre os gêneros e pela dignidade no cuidado das pessoas de todas as idades^23^.

Um ponto-chave do PMC é sua intersetorialidade que conecta diferentes atores e equipes multidisciplinares e permeia todas as operações do Programa: reuniões conjuntas para revisão de casos, contribuições combinadas em planos de cuidados individualizados e comunicação contínua com os cuidadores^6^. Assim, o Programa insere-se nas redes de atenção, sobretudo, da atenção primária – nível prioritário para assistir e monitorar o estado de saúde da população idosa^25^.

Outro elemento importante interligado à atuação dos cuidadores sociais no Programa é a inclusão social deles pelo trabalho formal. Com o salário, vem também a definição de funções específicas e responsabilidades claras, incluindo o tempo de trabalho em cada domicílio. Experiências com cuidadores voluntários em países como Costa Rica e Tailândia mostram que, embora estes forneçam algum apoio, suas contribuições são limitadas e inconsistentes^26^.

O Programa atua em comunidades que enfrentam problemas e privações das mais diversas ordens, mas uma questão central do PMC é não se restringir à saúde físico-funcional de cada idoso, mas considerar o contexto situacional para oferecer estratégias mais abrangentes de apoio, realizar o acompanhamento direto dos idosos assistidos e alcançar as demais pessoas e políticas envolvidas, para além do domicílio. No universo pesquisado, para os diferentes atores é uníssona a percepção de que o Programa é muito importante e apresenta estratégias e implicações positivas para diferentes eixos de atuação. Seus objetivos e ações são claros, entretanto persistem lacunas a serem rediscutidas nos cenários de atuação para que o Programa tenha processos e resultados cada vez mais exitosos.

## CONCLUSÃO

Este estudo realizou uma análise qualitativa do PMC – estratégia de ação intersetorial que oferta cuidados a idosos em situações de alta vulnerabilidade clínica e social em Belo Horizonte. O Programa, enquanto uma inovação social, oferece a oportunidade aos participantes de desenvolver uma velhice digna, com suporte em atividades e alívio para a sobrecarga de trabalho dos cuidadores familiares. Com ações e orientações básicas de cuidado, ele se mostrou uma iniciativa capaz de minimizar a ocorrência de agravos em saúde, de modo a evitar hospitalizações e institucionalizações.

Este artigo tem como principal limitação as dificuldades encontradas durante a coleta de dados devido à falta de registro e sistematizações nas regionais de saúde que aderiram ao PMC, situação que vem sendo corrigida a partir da própria pesquisa.

Nesse contexto de envelhecimento, a escuta dos atores envolvidos no PMC possibilitou um entendimento ampliado das múltiplas questões envolvidas no cuidado e nas políticas públicas, especialmente em áreas de maior vulnerabilidade social, como é o lócus de atuação do PMC. Espera-se que esta pesquisa levante novas discussões e estratégias que contribuam para o fortalecimento do Programa e construção de políticas de cuidado à pessoa idosa.
